# Comparative Efficacy of Once-Weekly Somatrogon Versus Daily Growth Hormone Therapy in Children With Idiopathic Growth Hormone Deficiency: A Real-World Retrospective Study From Greece

**DOI:** 10.7759/cureus.82998

**Published:** 2025-04-25

**Authors:** Sokratis Katsoudas, Evangelia Tsitsekli, Ioannis Pichlinski, Nikolitsa Techlemetzi, Eleni Galanopoulou, Ioulia Polychroni, Paraskevi Zosi

**Affiliations:** 1 Pediatrics, General Hospital of Nikaia Peiraia Agios Panteleimon, Nikaia, GRC; 2 Pediatric Endocrinology, Private Unit, Athens, GRC; 3 Pediatric Endocrinology, General Hospital of Nikaia Peiraia Agios Panteleimon, Nikaia, GRC

**Keywords:** gh, ghd, gh therapy, growth hormone, growth hormone deficiency, short stature, somatrogon, weekly gh therapy

## Abstract

Introduction

Short stature is one of the most common reasons for referral to a pediatric endocrinologist, defined as a height of at least two standard deviations (SD) below the mean for age and sex. In this study, we aimed to compare the efficacy of daily versus weekly growth hormone (GH) treatments on the growth of children with idiopathic growth hormone deficiency (IGHD).

Methods

In this six-month study, participants were matched 1:1 between once-weekly somatrogon and once-daily GH treatment cohorts. A total of 20 children (aged 4-15 years) with IGHD who had never received GH treatment were included.

Results

Height gain at six months was 4.58 ± 1.18 cm for somatrogon-treated (weekly) subjects and 4.41 ± 0.87 cm for subjects receiving the daily regimen. Changes in height standard deviation score (SDS) by six months were similar in both treatment groups. Both treatments were well tolerated.

Conclusion

Both daily and weekly GH regimens significantly increased height and height SDS, with similar efficacy and safety profiles, over the six-month period.

## Introduction

Short stature is one of the most common causes of referral to a pediatric endocrinologist and is defined as a height of at least two standard deviations (SD) below the mean for age and sex [1-3]. Common normal variants of short stature include familial short stature, constitutional delay of growth and puberty, and idiopathic short stature. Pathologic causes of short stature include chronic diseases and genetic disorders (such as Turner syndrome), as well as growth hormone deficiency (GHD) [4].

GHD, diagnosed during childhood or adolescence, may present as an isolated deficiency or as part of a combined pituitary hormone deficiency. The severity can range from severe GHD (peak growth hormone (GH) < 3 ng/mL on stimulation testing) to partial GHD (peak GH 3-7 ng/mL) [5]. GHD can result from idiopathic, genetic, or acquired causes; however, the most common cause in children is idiopathic [6]. GH replacement therapy has consistently been proven effective in helping patients reach an adult height within their genetically predicted range, especially when treatment begins at a younger age [7].

Until recently, GH treatment required once-daily injections at a specific time each day, creating a strict routine that could be burdensome for children and their parents. This need for flexibility is especially pronounced in our country, where adolescents’ lifestyles demand more adaptable regimens. Furthermore, studies indicate that treatment adherence tends to decline over time [8,9], highlighting the need for a more adaptable approach to GH therapy. In this study, we aimed to compare and evaluate the efficacy of daily GH versus weekly somatrogon administration in 20 treatment-naive patients with idiopathic GHD.

History of GH treatment

Human GH, also known as somatotropin, is a polypeptide of 191 amino acids produced by the anterior pituitary gland [10]. This hormone plays a critical role in growth both directly (promoting growth in nearly all tissues and organs, especially during adolescence) and indirectly (stimulating hepatic insulin-like growth factor-1 (IGF-1) secretion) [10].

In 1956, bovine-derived GH was used therapeutically for the first time. By 1959, patients with GHD were receiving replacement therapy using human-derived GH [11]. However, in 1985, the use of cadaveric human GH was halted due to its association with Creutzfeldt-Jakob disease in recipients. That same year, the Food and Drug Administration (FDA) approved recombinant human GH for pediatric GHD patients [11,12]. Over time, the therapeutic use of recombinant human GH (rhGH) was approved for additional conditions such as chronic renal insufficiency, adult GHD, Turner syndrome, idiopathic short stature, and Noonan syndrome, among others [12]. Because rhGH therapy requires daily long-term administration and its effectiveness depends on patient adherence, a need arose for the development of a long-acting formulation [13]. The first long-acting GH (LAGH) product was approved in 1999 but was withdrawn in 2004. Subsequent advancements include approvals of LAGH products in China, South Korea, the USA, and other markets, with the most recent being somatrogon in 2022 for pediatric GHD in multiple countries [14].

Mechanism of action

Somatrogon is a fusion protein produced in hamster ovary cells via recombinant DNA technology. It is composed of the amino acid sequence of human GH with one copy of the C-terminal peptide (CTP) from the β-chain of human chorionic gonadotropin (β-hCG) fused to the GH at the N-terminus, and two copies of CTP at the C-terminus [15,16].

Somatrogon-ghla binds to GH receptors, leading to the activation of the STAT5b signaling pathway and an increase in serum IGF-1 levels. Following subcutaneous injection, serum somatrogon concentrations rise slowly, peaking approximately 11 hours post-dose [16]. After multiple doses, IGF-1 standard deviation score (SDS) values remain in the normal range for pediatric patients. IGF-1 levels peak roughly two days post-dose, with the average weekly IGF-1 level occurring around four days after injection [16].

The molecular structure of somatrogon results in slower renal clearance, extending the half-life from about four hours for somatropin to ~37.7 hours for somatrogon [16,17]. Consequently, somatrogon remains in circulation for up to eight days after administration [16,17].

## Materials and methods

This retrospective, real-world comparative study evaluated the efficacy of weekly somatrogon versus daily GH therapy in pediatric patients with idiopathic GHD. Data were obtained from a private pediatric endocrinology unit owned and operated by Dr. Ioulia Polychroni in Athens, Greece, by reviewing patient files and selecting those that met the inclusion criteria. Informed consent was obtained from the caregivers of the patients. Patients' allocation to either regimen was determined by the historical availability of the treatment and caregiver choice following an in-depth discussion of the risks and benefits associated with each GH regimen.

Population

The study included male and female patients aged from four to 15 years at the beginning of treatment who were diagnosed with idiopathic GHD.

Inclusion criteria

Patients included in the study were those aged between four and 15 years, diagnosed with idiopathic GHD, and without any comorbidities affecting growth. Only patients who had never been treated previously for GHD were considered, and all patients were in Tanner stages I-II. Additionally, complete biochemical and anthropometric data were available at baseline and six months after treatment.

Exclusion criteria

Patients were excluded if they were not within the 4-15 years age range, if their GHD had a known cause other than idiopathic, if they were born small for gestational age, if they had received prior treatment for GHD, or if their anthropometric and biochemical data were incomplete either at baseline or at the six-month follow-up.

Every patient from the weekly cohort was matched 1:1 to a patient from the daily cohort in an effort to eliminate confounding factors. Each pair was matched for the same sex, age (within a five-month range), initial height (within a 5 cm range), initial BMI (within a range of ±1.5), initial pubertal status (Tanner stages I-II), maximum GH peak at provocative tests (within a one-unit range), initial IGF-1 levels (within a range of ±100), and baseline bone age (within a 0.5-year range).

Intervention

The weekly cohort patients received once-weekly somatrogon GH treatment with a dosage of 0.66mg/kg per week, while the daily cohort patients received daily GH treatment with a mean dosage of 0.23mg/kg per week divided into 6-7 doses. 

Outcomes

Biochemical and anthropometric data were measured at baseline and again six months after the initiation of GH therapy. The primary outcomes assessed were the change in height (in cm) and the change in height velocity (in cm per year). The secondary outcomes included the change in IGF-1 levels, weight gain, and progression of bone age.

Measurement standardization

To ensure consistency across the study, height was measured using the same stadiometer (Hyssna Limfog AB, Sweden) for all patients, and weight was recorded with the same calibrated scale (SECA Model 877, SECA GmbH & Co. KG, Hamburg, Germany). Biochemical tests, including IGF-1, were analyzed in the same laboratory, and bone age radiographs were evaluated by the same experienced pediatric endocrinologist using the Greulich and Pyle Atlas. Serum IGF-1 was measured via chemiluminescent immunoassay on an automated immunoanalyzer (IMMULITE 2000 Immunoassay System, Siemens Healthineers, Erlangen, Germany).

Statistical analysis

For descriptive statistics, quantitative variables were expressed as mean ± SD if normally distributed, or as medians with interquartile ranges if not. Qualitative variables were expressed as frequencies (percentages). The normality of the distribution was checked using the Shapiro-Wilk test due to the small sample size (n < 30). Comparisons between the daily and weekly dosing groups were performed using the parametric Student’s t-test, as most variables followed a normal distribution, with the exception of the change in bone age (Δbone age), which was analyzed using the non-parametric Mann-Whitney test. Time comparisons within each dosing group (baseline versus six-month follow-up) were carried out using the paired t-test, and the Chi-squared dependence test was used to compare categorical variables. All statistical tests were two-tailed with a significance level set at α = 0.05. The analysis was performed using IBM SPSS Statistics for Windows, version 27 (IBM Corp., Armonk, USA).

## Results

Baseline characteristics

A total of 20 children (10 in the daily growth hormone group and 10 in the weekly group) were included in the analysis. The two groups were similar in terms of sex distribution, age, height, BMI, height SDS, height velocity, pubertal status, bone age, GH peak value at the provocative tests, and IGF-1 levels at baseline (all p > 0.05). The results, as depicted in Table 1, demonstrate that the two groups were well-matched at baseline.

**Table 1:** Summary of baseline characteristics for the daily and weekly GH treatment groups. GH: Growth hormone; IGF-1: Insulin-like growth factor-1; BMI: Body mass index; SDS: Standard deviation score

Variable	Daily (n = 10)	Weekly (n = 10)	t/χ² statistic	p-value	Effect size (Cohen’s d/φ) – 95% CI of the mean difference
Sex (male/female)	8 (80%) / 2 (20%)	8 (80%) / 2 (20%)	χ²(1)=0.00	1	φ = 0.00
Age (years)	8.78 ± 2.80	9.14 ± 3.42	t(18)=-0.26	0.796	3.12 (-3.30 – 2.57)
Height (cm)	118.3 ± 15.2	119.8 ± 17.5	t(18)=-0.20	0.842	16.4 (-16.89 – 13.93)
BMI (kg/m²)	15.8 ± 1.2	17.7 ± 2.8	t(18)=-2.00	0.061	2.17 (-4.00 – 0.10)
Height SDS (cm)	-2.21 ± 0.35	-2.30 ± 0.46	t(18)=0.45	0.662	0.41 (-0.31 – 0.47)
Height velocity (cm/year)	2.7 ± 0.4	2.8 ± 0.4	t(18)=-0.302	0.766	0.37 (-0.40 – 0.30)
Pubertal status (Ι/ΙΙ)	8 (80%) / 2 (20%)	6 (60%) / 4 (40%)	χ²(1)=0.952	0.329	φ = 0.22
Bone age (years)	7.24 ± 2.91	7.17 ± 3.48	t(18)=0.05	0.959	3.21 (-2.94 – 3.09)
GH peak value (ng/mL)	6.43 ± 1.56	5.48 ± 2.15	t(18)=1.13	0.272	1.88 (-0.81 – 2.72)
IGF-1 (ng/mL)	104.4 ± 41.3	122.3 ± 57.6	t(18)=-0.798	0.435	50.15 (-65.02 – 29.22)

Between-group comparisons of the six-month follow-up

**Figure 1:**
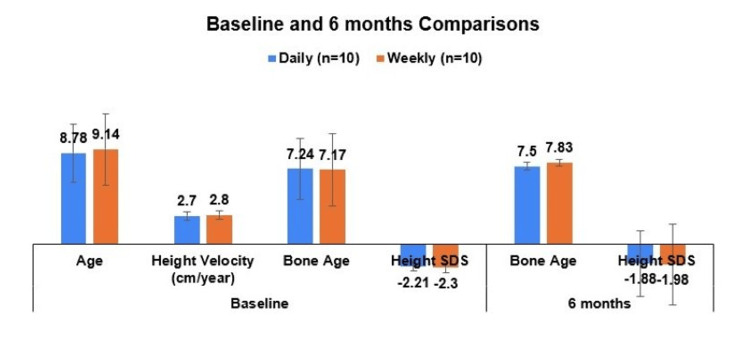
Comparison of age and height velocity between the daily and weekly GH treatment groups at baseline, as well as bone age and height SDS at baseline and six months. GH: Growth hormone; SDS: Standard deviation score

Similar comparisons were made for the six-month follow-up period in the basic parameters measured between the daily and weekly dosing groups. No statistically significant differences were observed in height, BMI, height SDS, bone age, and IGF-1, though IGF-1 was numerically higher in the weekly group (Table 2, Figure 1). 

**Table 2:** Summary of comparisons between the daily and weekly GH treatment groups in key parameters at six-month follow-up. GH: Growth hormone; IGF-1: Insulin-like growth factor-1; BMI: Body mass index; SDS: Standard deviation score

Variable	Daily (n = 10)	Weekly (n = 10)	t statistic	p-value	Effect size (Cohen’s d) – 95% CI of the mean difference
Height (cm)	122.7 ± 15.1	124.4 ± 18	t(18)=-0.22	0.827	16.62 (-17.26 – 13.96)
BMI (kg/m²)	16.4 ± 1.5	18.4 ± 3	t(18)=-1.88	0.076	2.4 (-4.27 – 0.23)
Height SDS (cm)	-1.88 ± 0.36	-1.98 ± 0.36	t(18)=0.61	0.549	0.36 (-0.24 – 0.44)
Bone age (years)	7.5 ± 3.17	7.83 ± 3.94	t(16)=-0.20	0.845	3.53 (-3.88 – 3.21)
IGF-1 (ng/mL)	185.4 ± 63.8	254.7 ± 145	t(12.362)=-1.38	0.191	111.99 (-178.07 – 39.47)

Within-group changes (baseline to six-month follow-up)

**Figure 2:**
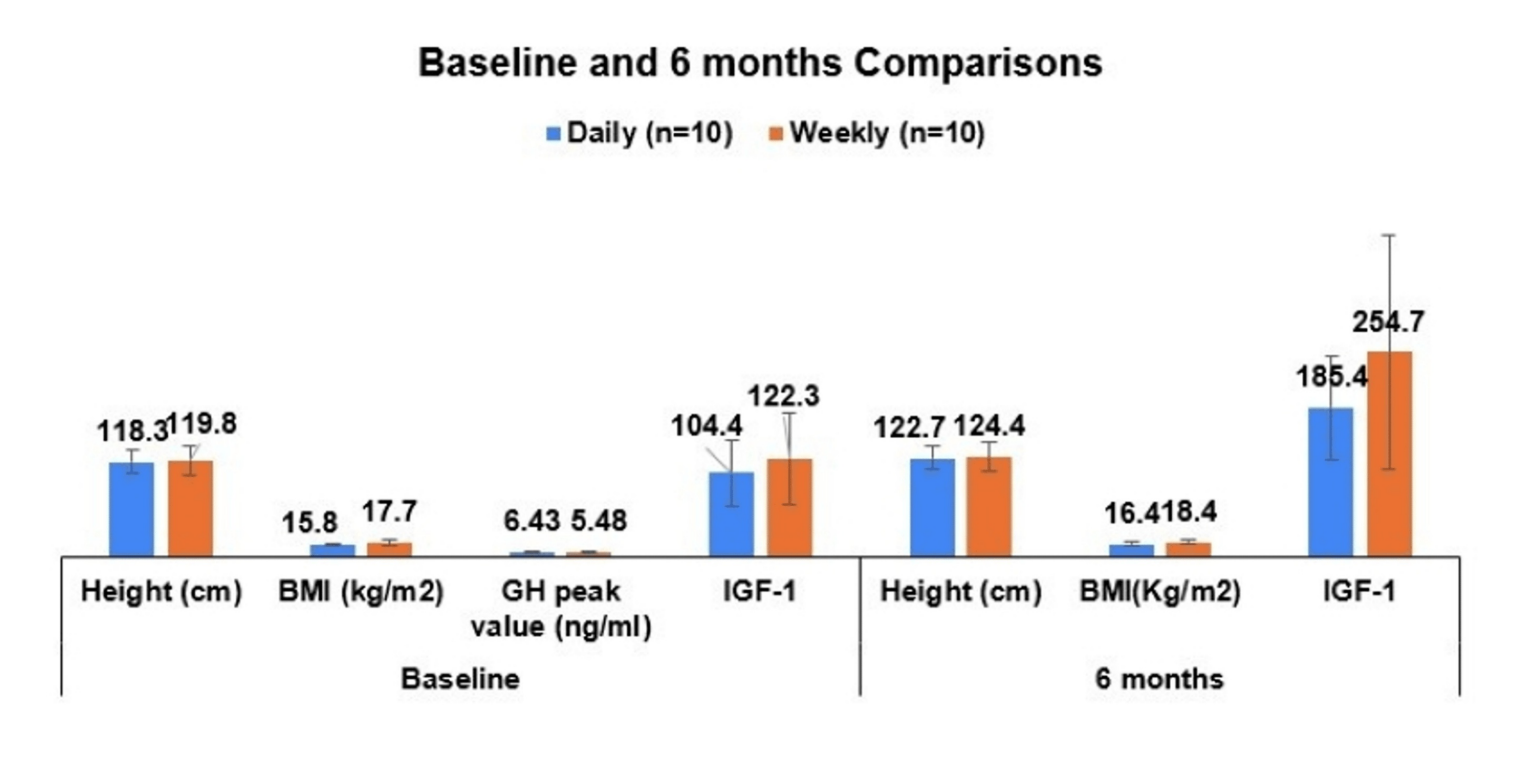
Comparison of height, BMI, and IGF-1 levels between the daily and weekly GH treatment groups at baseline and at six-month follow-up, along with mean GH peak values from stimulation tests for both groups. GH: Growth hormone; IGF-1: Insulin-like growth factor-1; BMI: Body mass index

**Figure 3:**
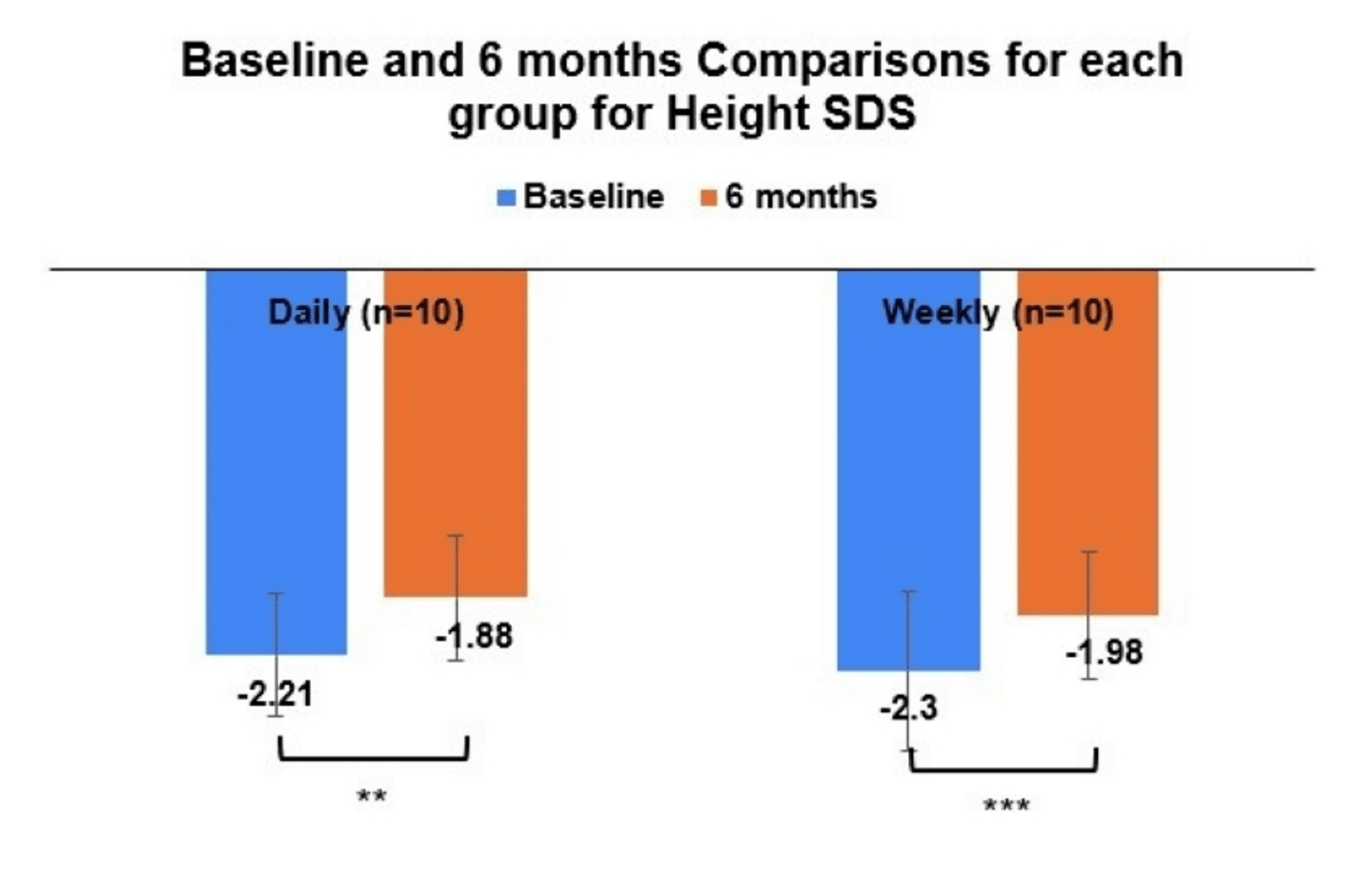
Height SDS in the daily and weekly GH treatment groups at baseline and at six-month follow-up. GH: Growth hormone; SDS: Standard deviation score ** indicates p < 0.01; *** indicates p < 0.001

Within-group comparisons were conducted to examine how height, BMI, height SDS, bone age, and IGF-1 changed over the six-month treatment period. Participants receiving daily GH showed significant increases in height, BMI, height SDS, bone age, and IGF-1 levels (Table 3, Figure 2). More specifically, height increased significantly from baseline (Mean = 118.3 cm, SD = 15.2) to six months (Mean = 122.7 cm, SD = 15.1), t(9) = -15.99, p < 0.001, d = 0.87. BMI increased modestly (t(9) = -2.3, p = 0.047, d = 0.83) from baseline (Mean = 15.8 kg/m², SD = 1.2) to six months (Mean = 16.4 kg/m², SD = 1.5). Height SDS improved significantly (t(9) = -3.25, p = 0.01, d = 0.32) from baseline (Mean = -2.21 cm, SD = 0.35) to six months (Mean = -1.88 cm, SD = 0.36) (Figure 3). IGF-1 levels showed a large and significant increase (t(9) = -6.2, p < 0.001, d = 41.36) from baseline (Mean = 101.4 ng/mL, SD = 41.3) to six months (Mean = 185.4 ng/mL, SD = 63.8), confirming a strong response to GH treatment. A marginally non-statistically significant increase was also observed for bone age (t(9) = -2.26, p = 0.05, d = 0.36) from baseline (Mean = 7.24 years, SD = 2.91) to six months (Mean = 7.83 years, SD = 3.94).

**Table 3:** Within-group changes in key growth parameters over six months. GH: Growth hormone; IGF-1: Insulin-like growth factor-1; BMI: Body mass index; SDS: Standard deviation score * indicates p < 0.05; ** indicates p < 0.01; *** indicates p < 0.001

Variable	0 months (mean ± SD)	6 months (mean ± SD)	t statistic	p-value	Effect size (Cohen’s d) – 95% CI of the mean difference
Daily GH group					
Height (cm)	118.3 ± 15.2	122.7 ± 15.1	t(9)=-15.99	<0.001	0.87*** (-5.03 – -3.79)
BMI (kg/m²)	15.8 ± 1.2	16.4 ± 1.5	t(9)=-2.3	0.047	0.83* (-1.19 – -0.01)
Height SDS (cm)	-2.21 ± 0.35	-1.88 ± 0.36	t(9)=-3.25	0.01	0.32** (-0.56 – -0.10)
Bone age (years)	7.24 ± 2.91	7.5 ± 3.17	t(9)=-2.26	0.05	0.36 (-0.52 – -0.0001)
IGF-1 (ng/mL)	104.4 ± 41.3	185.4 ± 63.8	t(9)=-6.2	<0.001	41.36*** (-110.62 – -51.44)
Weekly GH group					
Height (cm)	119.8 ± 17.5	124.4 ± 18.0	t(9)=-12.3	<0.001	1.18*** (-5.42 – -3.74)
BMI (kg/m²)	18.7 ± 3.8	18.4 ± 3.0	t(9)=0.26	0.799	3.01 (-1.90 – 2.40)
Height SDS (cm)	-2.30 ± 0.46	-1.98 ± 0.36	t(9)=-5.40	<0.001	0.18*** (-0.44 – -0.18)
Bone age (years)	7.17 ± 3.48	7.83 ± 3.94	t(7)=-1.727	0.128	0.72 (-1.03 – 0.16)
IGF-1 (ng/mL)	122.3 ± 57.6	254.7 ± 145.0	t(9)=-3.83	0.004	109.37** (-210.67 – -54.19)

Similarly, participants receiving weekly GH also experienced significant height, height SDS, and IGF-1 increases, but without significant changes in BMI or bone age. Height increased significantly (t(9) = -12.3, p < 0.001, d = 1.18) from baseline (Mean = 119.8 cm, SD = 17.5) to six months (Mean = 124.4 cm, SD = 18.0). Height SDS improved significantly (t(9) = -5.40, p < 0.001, d = 0.18) from baseline (Mean = -2.30 cm, SD = 0.46) to six months (Mean = -1.98 cm, SD = 0.36). IGF-1 levels increased significantly (t(9) = -3.83, p = 0.004, d = 109.37) from baseline (Mean = 122.3 ng/mL, SD = 57.6) to six months (Mean = 254.7 ng/mL, SD = 145.0), suggesting a strong GH effect despite less frequent injections. Unlike the daily group, BMI did not change significantly (p = 0.799) and nor did bone age (p = 0.128).

Between-group comparisons of changes

To evaluate differences in treatment effects between daily and weekly GH groups, the change from 0 to six months (Δ) was compared. The corresponding variables were recalculated as the difference between the value at six months minus the value at baseline (Table 4).

**Table 4:** Between-group comparisons of changes (Δ) over six months. GH: Growth hormone; IGF-1: Insulin-like growth factor-1; BMI: Body mass index; SDS: Standard deviation score

Variable	Daily (mean ± SD)	Weekly (mean ± SD)	t/U statistic	p-value	Effect size (Cohen’s d/ r) – 95% CI of the mean difference
ΔHeight (cm)	4.41 ± 0.87	4.58 ± 1.18	-0.37	0.718	1.04 (-1.14 – 0.80)
ΔBMI (kg/m²)	0.6 ± 0.83	0.67 ± 0.93	-0.18	0.860	0.88 (-0.89 – 0.75)
ΔHeight SDS (cm)	0.33 ± 0.32	0.31 ± 0.18	0.15	0.886	0.26 (-0.23 – 0.26)
ΔIGF-1 (ng/mL)	81.03 ± 41.4	132.43 ± 109.4	-1.39	0.181	82.68 (-129.09 – 26.29)
ΔBone age (years)	0 (0.52)	0 (0.75)	U=37	0.761	r=0.107 (-0.10 – -0.05)

No statistically significant differences were observed in Δheight, ΔBMI, Δheight SDS, or Δbone age (all p > 0.05), though ΔIGF-1 was numerically higher in the weekly group (p = 0.181).

Both daily and weekly GH treatments were equally effective in increasing height and growth rate, with no significant difference in height gain (p = 0.718) or height SDS improvement (p = 0.886) between the two groups. While IGF-1 levels increased more in the weekly GH group, this difference was not statistically significant (p = 0.181), though the large effect size suggests a potential trend favouring weekly GH. Weekly GH showed a trend toward greater weight gain, as BMI increased in the weekly group; however, this difference was not statistically significant (p = 0.860). Finally, bone age progression was similar in both groups (p = 0.761), indicating that neither treatment accelerated skeletal maturation more than the other.

## Discussion

The objective of this study was to evaluate the efficacy of weekly somatrogon compared to once-daily GH treatment in children with GHD. Notably, to our knowledge, this is the first real-world dataset from Greece regarding somatrogon use, providing valuable insights into its clinical application. The goal was to assess differences in height gain, IGF-1 changes, and bone age advancement between weekly somatrogon and daily therapy.

Efficacy

In the present study, both weekly and daily GH treatments significantly improved linear growth and IGF-1 levels in children with GHD. Height increased substantially in both groups over six months, with no statistically significant difference in absolute height gain or height SDS improvement between the weekly and daily regimens. The mean height gain was 4.4 cm in the daily group and 4.6 cm in the weekly group, reflecting comparable efficacy. Similarly, height SDS improved from -2.21 to -1.88 in the daily group and from -2.30 to -1.98 in the weekly group, demonstrating equivalent growth. These findings indicate that once-weekly GH treatment provides a growth response comparable to that of daily GH treatment, supporting its use as an alternative therapeutic option. Although our study, with a limited sample size, did not show one treatment to be statistically superior to the other, numerous studies have established the non-inferiority of weekly GH dosing compared to daily dosing [15,16,18].

Both treatment groups exhibited significant increases in IGF-1 levels over the six-month period. The weekly GH group showed a larger rise in IGF-1 levels, possibly because some patients had measurements taken before 96 hours post-dose, which may capture peak levels rather than the mean levels, as discussed below. Specifically, in our study, the daily group’s IGF-1 rose from ~104 ng/mL at baseline to ~185 ng/mL at six months, whereas the weekly group’s IGF-1 rose from ~122 ng/mL to ~255 ng/mL. While we did not find a statistically significant difference between groups in IGF-1 increase (likely due to variability and sample size), the trend aligns with the phase 3 trial showing higher IGF-1 responses with weekly GH, which was also partially attributed to measurements before the 96-hour limit [15].

It is important to consider the timing of IGF-1 measurement in interpreting these results. In daily GH administration, IGF-1 levels remain fairly stable throughout the day, so timing is less critical. However, in weekly GH therapy, IGF-1 levels vary depending on how long after the injection the measurement is taken [19,20]. Mean levels of IGF-1 are captured at around 96 hours post-dose, but measuring IGF-1 earlier (around 48-72 hours post-injection) would capture the peak IGF-1 level [15]. Although there is no clear evidence that IGF-1 values above +2 SDS increase adverse event risk, keeping IGF-1 within -2 to +2 SDS is generally recommended for safety and efficacy optimization [17]. In our study, one patient in the weekly cohort required a dose reduction after one month of treatment due to elevated IGF-1 levels. The dose was decreased by 15%, which subsequently normalized the IGF-1 levels. This dose adjustment is consistent with protocols used in pivotal phase III clinical trials, where dose reductions of 15-20% were implemented when average IGF-1 levels exceeded +2 SDS [21].

Bone age increased in both treatment groups over six months (from a mean of 7.24 to 7.50 years in the daily group, and 7.17 to 7.83 years in the weekly group). This increment was proportional to chronological time and did not significantly differ between the groups. Importantly, the bone age/chronological age ratio remained below 1.0 in both cohorts after six months, indicating that neither weekly nor daily GH therapy caused excessive acceleration of skeletal maturation.

Safety

In this study, the safety profiles of LAGH (weekly somatrogon) and daily rhGH appeared similar, consistent with published data [22]. The most common adverse events were mild injection-site reactions and pain, which were reported more frequently with the weekly injections. However, these effects were mild, and no patient discontinued treatment due to adverse events. This suggests that weekly GH therapy was generally well tolerated over the short-term treatment period, with no new or unexpected safety concerns.

Adherence

Poor adherence to daily GH injections is known to adversely affect growth outcomes [23]. In our retrospective study, we could not directly measure adherence. However, other studies have documented that adherence to GH therapy tends to decline over time with daily regimens. For example, the Easypod Connect Observational Study (ECOS) by Koledova et al., which tracked pediatric patients in 24 countries on daily GH via an electronic autoinjector, found that adherence was initially high (93.7% in the first year) but dropped to about 70% after five years [24]. Notably, that study demonstrated a significant correlation between adherence and growth outcomes, underscoring that suboptimal adherence can substantially impair the effectiveness of GH therapy [24].

Weekly GH injections offer a potential advantage in this context by reducing the treatment burden and possibly improving adherence. Although we did not measure adherence, the comparable growth results achieved with weekly therapy in our study suggest that if adherence to daily therapy were suboptimal, switching to a weekly regimen might mitigate some of the negative impact of missed doses.

Limitations

This study has several limitations. The small sample size (20 patients) limits the generalizability of the findings; larger, randomized studies are needed to confirm these results. Additionally, we did not directly monitor adherence in our cohort. Future research should incorporate objective adherence tracking (e.g., electronic injection devices) to better evaluate how adherence differences might influence outcomes with weekly vs. daily regimens. Moreover, our follow-up duration was relatively short (six months). Long-term studies are necessary to determine whether weekly GH therapy affects final adult height, metabolic parameters, or other long-term outcomes differently than daily therapy. Lastly, as a retrospective study, there was an inherent risk of selection bias. However, this was minimized by 1:1 matching and the demonstration that baseline characteristics between the two groups were statistically comparable.

Our experience

Existing literature suggests that weekly GH therapy is an effective option that can offer significant benefits for pediatric patients [15,18]. However, given its relatively recent introduction, long-term clinical experience with weekly GH remains limited. In our view, weekly GH therapy should be considered primarily for children who have difficulty adhering to daily injections, or whose quality of life (or that of their family) is significantly affected by the burden of daily shots.

In our clinical experience, adolescents tend to prefer weekly GH therapy due to the reduced injection frequency and greater convenience. We have also observed that some children with poor adherence to daily GH showed improved growth velocity after switching to weekly injections. This anecdotal experience suggests that, for select patients, the flexibility of weekly dosing can translate into better real-world effectiveness. Remarkably, preliminary results from a pilot study that we are currently conducting, of patients that transitioned from the daily to the weekly regimen, indicate that 94.1% of the patients showed preference for the weekly regimen, while 82.6% of the parents showed preference for the weekly regimen (Poster: Katsoudas S, Pichlinski I, Tsitsekli E, et al. Transitioning from Daily to Weekly Growth Hormone Therapy in Pediatric Idiopathic Growth Hormone Deficiency: Efficacy and Quality-of-Life Perspectives. Joint Congress of the European Society for Pediatric Endocrinology (ESPE) and the European Society of Endocrinology (ESE); May 10-13, 2025). These promising findings represent early unpublished data and require further validation, as additional questionnaires from a larger cohort of patients and their families are still pending. It is essential that patients and their caregivers make the final choice of treatment regimen, following a thorough discussion of the risks and benefits of each option.

An interesting question for future research is defining an adherence “threshold” beyond which a switch to weekly GH therapy yields better outcomes. Identifying a specific cutoff for what constitutes “poor adherence” (for example, missing a certain percentage of daily doses) that would justify transitioning to weekly injections could guide clinicians in personalizing treatment. A dedicated study to explore this threshold could provide valuable guidance for optimizing therapy in patients struggling with daily injections.

## Conclusions

In conclusion, once-weekly GH treatment with somatrogon demonstrated efficacy and safety comparable to the traditional once-daily GH regimen in children with GHD over a six-month period. Both regimens produced significant improvements in growth parameters with similar short-term safety profiles. These findings support somatrogon's use as a practical and effective alternative in real-world clinical settings. The added convenience and potential for improved adherence with weekly dosing may offer meaningful benefits for patients and families. Future studies with larger cohorts and longer follow-up would help further define the long-term impact on outcomes such as final height and metabolic health.
